# Retrospective Study of the Impact of SARS-CoV-2 Infection on Asthma Control in Children

**DOI:** 10.3390/jcm14020356

**Published:** 2025-01-09

**Authors:** Jaqueline Abdul-Razzak, Mihaela Ionescu, Radu Diaconu, Alexandru Dan Popescu, Elena Carmen Niculescu, Mihai Gafencu, Ileana Octavia Petrescu, Cristina Elena Singer, Liliana Anghelina, Cristian Gheonea

**Affiliations:** 1 Doctoral School, University of Medicine and Pharmacy of Craiova, 200349 Craiova, Romania; jaquelineabdulrazzak90@gmail.com; 2 Department of Pediatrics “Mother and Child”, Faculty of Medicine, University of Medicine and Pharmacy of Craiova, 200349 Craiova, Romania; carmen.niculescu@umfcv.ro (E.C.N.); ileana.petrescu@umfcv.ro (I.O.P.); cristina.singer@umfcv.ro (C.E.S.); liliana.anghelina@umfcv.ro (L.A.); cristian.gheonea@umfcv.ro (C.G.); 3 Department of Medical Informatics, Faculty of Dental Medicine, University of Medicine and Pharmacy of Craiova, 200349 Craiova, Romania; 4 Department of Endodontics, Faculty of Dental Medicine, University of Medicine and Pharmacy of Craiova, 200349 Craiova, Romania; alexandrudanpopescu20@gmail.com; 5 Department of Pediatrics III, Faculty of Medicine, University of Medicine and Pharmacy “Victor Babeș” of Timișoara, 300041 Timișoara, Romania; mgafencu@umft.ro

**Keywords:** asthma, COVID-19, cough, nitric oxide, ventilatory function test

## Abstract

Asthmatic children who tested positive for COVID-19 experienced changes in lung function and persistent symptoms following SARS-CoV-2 infection, even for several months after diagnosis, and with the same features as in an acute phase. This study aimed to analyze a pediatric age group (between 0 and 17 years old) diagnosed with asthma, and SARS-CoV-2 infection attending regular monitoring visits in a Pediatric Department of a Regional Tertiary Hospital (Filantropia Clinical Municipal Hospital Craiova, Romania) during the COVID-19 pandemic and post-pandemic time interval (i.e., March 2020–July 2024), and identify how the infection influenced their long-term symptoms and treatment. **Materials and Methods**. The following variables were recorded: demographic data (gender, age group, residence), data related to allergies (allergic rhinitis, atopic dermatitis, and food allergies), the presence of exacerbations, the fraction of exhaled nitric oxide, the ventilatory function, the asthma phenotype (allergic or non-allergic), as well as the GINA assessment of asthma control at clinical visits were analyzed. SARS-CoV-2 infections were evaluated in terms of year of infection, symptoms, cough presence and persistence, and modifications of the asthma treatment during and after COVID-19 disease. The data were statistically analyzed with SPSS, using the Mann–Whitney U, Kruskal–Wallis H, and Chi-Square tests. **Results**. A lower incidence of COVID-19 cases was recorded in the first pandemic of asthmatic patients (2020 and 2021), but an increase in the rate of cases was observed at the beginning of the second pandemic, in 2022. The nitric oxide values in asthmatic children who were infected with SARS-CoV-2 were statistically significantly increased (*p* < 0.0005), especially for children with persistent cough for more than 4 weeks. A significant increase in the number of exacerbations was also observed in patients who tested positive for SARS-CoV-2 infection (*p* < 0.0005). Ventilatory function values were statistically significantly different in asthmatic children with and without SARS-CoV-2 infection (*p* < 0.05). **Conclusions**. The persistence of cough after the acute phase of SARS-CoV-2 infection as well as the changes in ventilatory tests emphasize the need of periodic medical check-ups, as well as the implementation of a therapeutic regimen appropriate for each pediatric patient.

## 1. Introduction

Asthma is one of the most important public health problems in the pediatric population in terms of chronic obstructive respiratory diseases [1,2]. This heterogeneous disease is characterized by the presence of symptoms that define chronic airway inflammation, namely coughing, wheezing, dyspnea, and chest tightness along with variable airflow limitation [3].

There are factors, called triggers, that cause the appearance of symptoms characteristic of asthma that set off different phenotypic types of asthma, forms that help establish appropriate therapeutic management. Nowadays, this chronic obstructive disease can be considered as a syndrome [4,5,6,7,8]. A common pediatric form is allergic-induced asthma associated with atopy (atopic dermatitis, allergic rhinitis) with an optimal and long-lasting response to inhaled corticosteroid therapy. Another phenotypic form is non-allergic asthma, a form without association with atopy, in which the response to inhaled corticosteroid therapy is not particularly long-lasting [3,8]. According to GINA, for patients diagnosed with severe asthma, there may be phenotype-based treatments available. However, for other patients, there is not yet a well-defined criterion to help in the good management of the disease, and more studies are needed to establish the potential link [3].

The World Health Organization (WHO) declared the onset of the COVID-19 pandemic in 2020 as requiring the implementation of strict safety measures to prevent its spread, such as hand washing, wearing masks in public places, closing crowded spaces (especially schools) and keeping optimal distance between people [9,10,11,12].

In the pediatric age group, the symptoms were mild, moderate or even asymptomatic, frequently affecting the airways, leading to cough, dyspnea, expectoration, and nasal congestion. Other common symptoms were fever, myalgia, arthralgia, headache, and gastrointestinal manifestations [13,14,15]. Although symptoms were similar to a seasonal cold during the acute phase of illness, the long-term effects in this age group were significant. The most common symptoms described in the literature were fatigability, respiratory symptoms (cough, dyspnea, chest tightness), difficulty concentrating, insomnia, anosmia, and ageusia [16,17,18].

Regarding the prevalence of COVID-19 disease, an increased number of hospitalizations was observed in asthmatic children who had contracted the SARS-CoV-2 virus, indicating a more severe evolution of the disease in pediatric asthmatic patients, but with a favorable outcome over time. Asthmatic children may be predisposed to the development of accentuated symptoms in severe forms of SARS-CoV-2 infection, with a cough being among the most frequent clinical signs in the acute period of the disease. An increased risk of severe COVID-19 disease was found in patients who received oral corticosteroids used to control asthma; the symptoms present in the case of exacerbations of children with asthma were more accentuated when they have contacted different infectious agents, especially the SARS-CoV-2 virus [19,20,21,22].

The Robert Koch Institute (RKI) defines long-COVID as a series of symptoms that persist for at least 4 weeks after the acute phase of the disease has ended [23]. The National Institute of Health (NIH) states that these symptoms persist for several months after patients have been diagnosed with COVID-19, presenting the same features as in the acute phase or even different symptoms [24]. The World Health Organization (WHO) based on the Delphi consensus, defines it as an onset of new symptoms three months after the acute phase of the disease with a duration of at least two months [25]. Defining long-COVID remains a challenge, but it is certain that the symptoms that occur after patients have been infected with SARS-CoV-2 affect daily life [16]. The effects of long-COVID in the pediatric age group with asthma are not fully understood, some authors consider that this pathology represents a breeding ground for the development of long-term sequelae [26,27].

The literature contains few data on the impact that SARS-CoV-2 infection has on pediatric patients who have been previously diagnosed with asthma. The novelty of the study was the recording and statistical analysis of important parameters regarding pulmonary ventilatory function such as FVC, FEV1, PEF, and FEF_25-75_ and exhaled nitric oxide in asthmatic children diagnosed with COVID-19, compared to those who have not contacted the virus but diagnosed with asthma, analyzing how COVID-19 disease may influence their symptomatology and treatment.

The present study aims to analyze the pediatric age group diagnosed with asthma, patients who tested positive for SARS-CoV-2 infection and how it influenced their long-term symptoms and treatment, by determining the respiratory function tests along with other parameters.

## 2. Materials and Methods

### 2.1. Study Design and Participants’ Selection

The retrospective clinical study followed a group of children previously diagnosed with asthma who were analyzed regarding potential infection with SARS-CoV-2 virus. The demographic data of the subjects (gender, age group, and residence), data related to allergies (allergic rhinitis, atopic dermatitis, and food allergies), as well as the presence of exacerbations, were recorded. Also, the fraction of exhaled nitric oxide, the ventilatory function, the asthma phenotype (allergic or non-allergic), as well as the GINA assessment of asthma control at clinical visits [3] were analyzed. The SARS-CoV-2 infections were evaluated in terms of year of infection, symptoms, cough presence and persistence, and modifications of the asthma treatment during and after COVID-19 disease.

The diagnostic devices used to determine the lung function of asthmatic children, consisted of the Vitalograph Pneumotrac 6800 spirometer (Vitalograph, Hamburg, Germany) and the Aerocrine Niox Vero 12-1000 analyzer (NIOX Group plc, Uppsala, Sweden).

The Vitalograph spirometer Pneumotrac 6800 is used in the acquisition of 4 parameters: FVC (forced vital capacity), FEV1 (forced expiratory volume in the first second), PEF (peak expiratory flow), and FEF_25-75_ (forced mid expiratory flow).

The Aerocrine Niox Vero 12-1000 analyzer measures the fraction of nitric oxide in exhaled air using a non-invasive method, the results of the measurements being molecularly expressed in ppb (parts per billion).

The study group included asthmatic children attending on regular monitoring visits in a Pediatric Department of a Regional Tertiary Hospital (Filantropia Clinical Municipal Hospital, Craiova, Romania) during the COVID-19 pandemic and post-pandemic time interval (i.e., March 2020–July 2024).

Review of medical records including data on acute COVID-19 disease were performed to meet inclusion and exclusion criteria. Inclusion criteria were as follows: (a) children under 18 years old whose parents or legal guardians have signed the informed consent necessary for this study; (b) children with known asthma under treatment; (c) positive SARS-CoV-2 infection through the PCR test or through antigen tests with clinical symptoms during the acute phase. Exclusion criteria were as follows: (a) children with asthma who present other chronic pathologies that may intervene in the results of the present study; (b) children with asthma confirmed with asymptomatic COVID-19 disease.

After analyzing the data from the medical records of pediatric asthmatic patients, 9 children who tested positive for the SARS-CoV-2 virus did not present symptoms during the acute period of COVID-19 disease or afterwards. This represented an exclusion criterion because they did not provide sufficient clinical information to help the development of the study.

**Figure 1:**
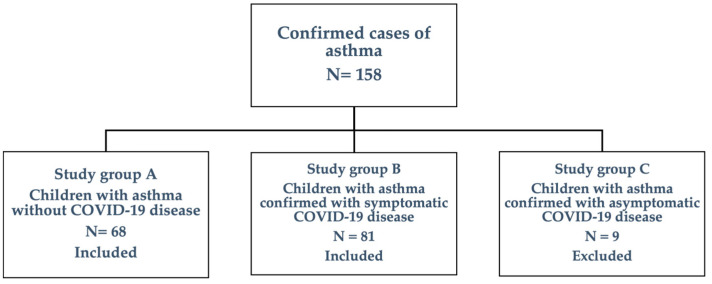
The structure of the study group.

Following the inclusion/exclusion criteria, a number of 149 children were finally enrolled in this study (56 girls and 93 boys) (Figure 1).

The study was approved by the Ethics Committee of the University of Medicine and Pharmacy of Craiova, no. 167/14.09.2023 and it respected the Declaration of Helsinki. All subjects’ parents or legal tutors signed an informed consent form on behalf of pediatric patients.

### 2.2. Statistical Analysis

The data collected from the patients’ charts were recorded using the Microsoft Excel 365 software application (Microsoft Corporation, Redmond, WA, USA). Data analysis was performed using the SPSS (Statistical Package for Social Sciences) software application, version 26 (SPSS Inc., Armonk, NY, USA) between May and November 2024. Continuous variables were described as mean ± standard deviation (SD), as well as median values. Nominal parameters were defined as frequency distributions and corresponding percentages. For all continuous variables, the Kolmogorov–Smirnov/Shapiro–Wilk test was used to assess the normality. According to the results obtained, comparisons between groups were performed using the Mann–Whitney U test or the Kruskal–Wallis H test. Associations between nominal variables were analyzed using the Chi-Square test. All *p* values smaller than 0.05 were considered statistically significant results.

## 3. Results

**Demographic data**

The study group included 149 asthmatic children, with the following gender distribution: (56 girls, representing 37.58% of the entire study lot, and 93 boys, 62.42%) (Table 1). There were no statistically significant differences in allergies, COVID-19 symptomatology, presence of cough, asthma exacerbations, nitric oxide, FEV1/FVC ratio parameters and GINA guideline assessment of asthma control at clinical visits by gender distribution in the study group. Most of the children originated from urban areas (107 children, representing 71.8% of the entire study group) and the rest from rural areas (42 children, representing 28.2%) (Table 1).

**Figure 2:**
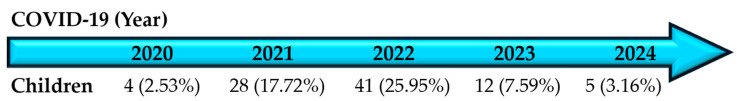
Time flow of SARS-CoV-2 infection diagnosis in the study group.

Between 2020 and 2024, 90 children diagnosed with asthma were confirmed with COVID-19, from which 81 patients had clinical symptoms during the acute phase. In this subgroup, children were counted only once, even if they had multiple positive diagnosis. Their distribution by year is presented in Figure 2.

Half of the children included in the study group have been diagnosed with asthma between 6 and 11 years old (75 children, representing 50.3%); 45% (67 children) had been diagnosed at early ages (less than 6 years old), and only 7 children (representing 4.7%) had been diagnosed as teenagers (ages above 12 years old) (Table 1).

Three quarters of children diagnosed with asthma at early ages associated allergic rhinitis (74.6%), compared to only 54.7% of children diagnosed at ages between 6 and 11 years old, and 42.9% of children diagnosed as teenagers. Thus, there was a statistically significant association between diagnosis age and the presence of allergic rhinitis, χ^2^(2) = 8.345, *p* = 0.025. The association was moderately strong Cramer’s V = 0.222.

FEV1/FVC ratio analysis: from the children diagnosed with asthma as teenagers, only 28.6% were included in the variable airflow limitation category, 65.3% of children diagnosed between 6 and 11 years old, and 74.6% of children diagnosed at early ages. Thus, there are statistically significant differences between the age of asthma diagnosis, relative to the FEV1/FVC ratio category, χ^2^(2) = 6.572, *p* = 0.037, Cramer’s V = 0.210. No other associations were identified.

**COVID-19 data analysis**

More than half of all children included in the study group were previously diagnosed with COVID-19 disease (81 children, representing 54.36% of the entire group). There were no statistically significant differences between gender, living areas or age group at asthma diagnosis regarding the infection with SARS-CoV-2 (Table 2).

There were no statistically significant associations between SARS-CoV-2 infections and allergies for the study group (Table 3). The analysis of risk factors for atopic manifestations, presented in Table 3, did not show a significant correlation with the clinical symptoms of the study group.

During the SARS-CoV-2 infection period, the parents of the affected children reported a series of symptoms (recorded only for the 81 children from COVID-19 subgroup). The distribution of symptoms is emphasized in Table 4.

Analysis of the number of exacerbations during the studied period is shown in Table 5. During the study period 109 children experienced exacerbations. Half of all children with COVID-19 suffered one exacerbation/year; almost a quarter suffered two exacerbations per year and only 8.64% suffered three exacerbations/year. The number of exacerbations for children without COVID-19 is lower and statistically significant, χ^2^(3) = 18.946, *p* < 0.0005, Cramer’s V = 0.357.

Similar results were obtained for compliance to controller therapy (parents/caregiver and/or self-reported), as only 34.57% of children with COVID-19 maintained their previous treatment, compared to 57.35% of children without COVID-19, χ^2^(1) = 7.755, *p* = 0.005, Cramer’s V = 0.228 (Table 5).

Less than half of all children without COVID-19 (44.12%) had a variable airflow limitation, compared to 87.65% of children with COVID-19, thus there is a statistically significant strong association between FEV1/FVC ratio and COVID-19 presence, χ^2^(1) = 32.087, *p* < 0.0005, Cramer’s V = 0.464 (Table 6).

Table 5 presents the correlation of level of asthma control with COVID-19 disease. The children with asthma without COVID-19, showed a better level of control, χ^2^(1) = 13.642, *p* < 0.0005, Cramer’s V = 0.303.

The asthma phenotype does not seem to be associated with COVID-19 disease, children with allergic asthma with COVID-19 disease (50.54%) had similar values with children with allergic asthma without COVID-19 (49.46%) (Table 6).

A Mann–Whitney U test was run to determine if there were differences in ventilatory function values between children with and without COVID-19. Distributions of the ventilatory function test variables for both categories of children were similar, as assessed by visual inspection.

As defined in Table 7, the median of exhaled nitric oxide for children with SARS-CoV-2 infection (30) was statistically significantly higher compared to children without previous SARS-CoV-2 infection (14.5), U = 1557.5, z = −4.168, *p* < 0.0005. Median FEF_25-75_ for children with SARS-CoV-2 infection (0.70) was statistically significantly lower compared to children without previous SARS-CoV-2 infection (0.82), U = 3340.5, z = 2.726, *p* = 0.006. Similarly, the median FEV1 and FEV1/FVC ratio for children with previous SARS-CoV-2 infection (0.78, respectively 0.88) was statistically significantly lower compared to children without previous SARS-CoV-2 infection (0.88, respectively 0.92), U = 3817.0, z = 4.397, *p* < 0.0005 for FEV1, and U = 3786.0, z = 4.497, *p* < 0.0005 for FEV1/FVC ratio.

On the other hand, median values for FVC and PEF were similar for children with and without previous SARS-CoV-2 infection, *p* > 0.05.

Since the most common symptom by parent perception in pediatric patients is cough, we studied its correlation with pulmonary function and inflammation of the airways (level of exhaled nitric oxide). Children were divided into three categories: cough that lasted less than 4 weeks, cough that lasted more than 4 weeks, and children who did not cough. There were no statistically significant differences between these groups regarding the demographic data, allergies, COVID-19 symptomatology, asthma treatment, exacerbations, asthma symptom control or asthma phenotype (*p* > 0.05).

Children in these categories were also analyzed with respect to the ventilatory function and exhaled nitric oxide (Table 8).

Children who were coughing for less than 4 weeks had reduced levels of exhaled nitric oxide. On the other hand, coughing for more than 4 weeks seemed to increase these parameters. Differences between groups were statistically significant for the nitric oxide, FEV1 and PEF (*p* < 0.05, Table 8).

## 4. Discussion

Some of the most important aspects of this study are the changes in lung function of asthmatic patients who tested positive for COVID-19 and the persistent symptoms following SARS-CoV-2 infection. Pediatric asthmatic patients are susceptible to viral infections increasing the risk of exacerbations, but a lower incidence has been observed especially during the pandemic, which explains the low number of cases available for inclusion in the present study, approximately one-third less than in the corresponding pre-pandemic time interval. Due to the safety measures, such as regular hand washing, wearing masks, keeping a safe distance between people, thus limiting contact with triggers, the rate of COVID-19 cases in asthmatic children has been lower [28,29,30,31,32,33,34]. The present study showed a lower incidence of COVID-19 cases in the first pandemic of asthmatic patients (2020 and 2021) with an increase in the rate of cases observed at the beginning of the second pandemic, i.e., in 2022.

Allergic rhinitis is a chronic inflammatory disease that affects the nasal mucosa and is mediated by IgE, affecting the pediatric population. Although the symptoms are minimal in children, such as rhinorrhea, nasal itching, and sneezing, it can cause fatigue, and learning and memory difficulty, affecting sleep and cognitive function [35,36,37,38,39]. Being an immunologically mediated disease with a genetic polymorphism, allergic rhinitis is accompanied by other diseases with similar substrates such as asthma and atopic dermatitis. Early-onset atopy is associated with the process of bronchial hyperreactivity and is a predictor of persistent asthma [40,41]. Throughout the pandemic, the presence of comorbidities was a risk factor in the development of long-COVID symptoms, such as obesity, allergic rhinitis, respiratory infections as well as symptoms during the acute phase of COVID-19 infection [42]. In the present study, atopic diseases were not directly linked to SARS-CoV-2 infection.

One of the natural defense mechanisms is cough which provides airway clearance and is also one of the most common symptoms during SARS-CoV-2 infection [43,44,45,46]. The symptoms reported in the pediatric age group during the acute phase of SARS-CoV-2 infection were cough with 55.56% followed by wheezing, pharyngodynia, and respiratory difficulty. It is known that in SARS-CoV-2 virus infection an inflammatory process occurs, an increase in mucus secretion and reactivity in the airways causing the appearance of cough reflex, being also one of the most common symptoms. Thus, patients with an atopic disease like asthma may suffer changes in the duration and persistence of cough after the acute phase of COVID-19 [47,48,49].

The control of asthma is associated with a reduction in the frequency and severity of exacerbations. Management of this disease is achieved with inhaled corticosteroid therapy, with patients or caregivers being required to adhere to the dose, number of administrations and correct administration techniques in order to achieve good disease control. During the pandemic, direct contact with the patient was affected, which led to a higher risk of instability and loss of control of chronic respiratory disease [50,51]. In this study, an increase in the number of exacerbations was observed in patients who tested positive for SARS-CoV-2 infection.

Measurement of nitric oxide levels is an important marker for the evolution of asthma in terms of adherence to inhaled corticosteroid therapy and possible exacerbations. These patients who are exposed to various infectious agents, such as SARS-CoV-2, have elevated exhaled nitric oxide levels. Values above 35 ppb indicate an eosinophilic type of inflammation requiring the adjustment of corticosteroid therapy [52,53,54,55,56]. According to the present study, the nitric oxide values in asthmatic children who were infected with SARS-CoV-2 were increased.

During the acute phase of infection symptoms are minimal or moderate in asthmatic patients but with future repercussions on lung function and symptomatology. The most sensitive spirometry parameter showing the degree of small airway obstruction is FEF_25-75_. Studies with low FEF_25-75_ values after COVID-19 infection have been reported as a significant indicator of an obstructive pattern [57,58,59,60,61].

One of the most commonly used devices to assess lung function in asthmatic children is the spirometer. SARS-CoV-2 infection has caused changes in the lung tissue, including the small airways, so that it can cause obstructive lung dysfunction. A child with a chronic obstructive lung disease who is exposed to SARS-CoV-2 infection may have spirometric changes. Low values of FVC and FEV1 have been reported after the acute infectious process has passed, explaining the remodeling process in the lung. Another valuable index in identifying changes occurring in the lung is the FEV1/FVC ratio which shows the level of airflow limitation, an index that also had low values after the acute infectious process [46,55,62,63,64]. The present study reported significant changes in patients with SARS-CoV-2 infection in both FEV1/FVC ratio and FEV1. Peak expiratory flow (PEF) is an indicator of pulmonary function assessment that provides important information about the airways and is determined before diagnosis and introduction of controller therapy in children with asthma. Studies in the literature have found a decrease in PEF values before the onset of exacerbations [65,66].

There are few studies showing whether asthma control has improved among the pediatric population during the pandemic, which may change the perception of the parents/caregivers about the controller medication [67,68].

### Limitations of the Study

Due to the unique characteristics of the pandemic, a relatively small number of asthmatic patients were reported in the early period of the pandemic (2020–2021), thus hampering quantitative data collection and also affecting the size of the study group. Another limitation of the study is the small number of pediatric asthmatic patients who tested positive for SARS-CoV-2 infection but were asymptomatic and could not be included in the study due to their low impact on the results of the present study.

The present study provides valuable information about the impact of SARS-CoV-2 infection on asthmatic children, further research should be conducted with a larger study group to more accurately assess the impact that COVID-19 disease had on children diagnosed with asthma.

## 5. Conclusions

The present research provides insights into the impact that COVID-19 has had on a pediatric population with chronic conditions, namely asthma, attending a Pediatric Department of a Regional Tertiary Hospital. The persistence of cough after the acute phase of SARS-CoV-2 infection as well as the changes in ventilatory tests highlight the need to insist on routine medical check-ups and the implementation of a therapeutic regimen appropriate for each pediatric patient. Understanding these factors would help to improve the quality of life of children with chronic obstructive diseases and reduce the sequelae of SARS-CoV-2 infection.

## Figures and Tables

**Table 1:** Distribution of the entire study group according to demographic data and the presence of allergies.

Parameter	Category	Value	N (%)/Mean ± SD
Demographic data	Gender	F	56 (37.58%)
		M	93 (62.42%)
	Environment	Urban	107 (71.81%)
		Rural	42 (28.19%)
	Age at asthma diagnosis	<6 years old	67 (44.97%)
		6–11 years old	75 (50.34%)
		12–17 years old	7 (4.7%)
Allergies	Allergic rhinitis	Present	94 (63.09%)
		Absent	55 (36.91%)
	Atopic dermatitis	Present	25 (16.78%)
		Absent	124 (83.22%)
	Food allergies	Present	13 (8.72%)
		Absent	136 (91.28%)
	Pollen	Present	29 (19.46%)
		Absent	120 (80.54%)
	House dust	Present	33 (22.15%)
		Absent	116 (77.85%)
	Animal hair	Present	20 (13.42%)
		Absent	129 (86.58%)
	Mold	Present	25 (16.78%)
		Absent	124 (83.22%)
	Tobacco	Present	3 (2.01%)
		Absent	146 (97.99%)

**Table 2:** Distribution of the study group according to demographic data.

Parameter	Category	COVID-19			*p* *
		Yes	No	Total	
		81 (54.36%)	68 (45.64%)	149 (100%)	
Gender	Females	32 (57.14%)	24 (42.86%)	56 (100%)	0.597
		39.51%	35.29%		
	Males	49 (52.69%)	44 (47.31%)	93 (100%)	
		60.49%	64.71%		
Environment	Urban	58 (54.21%)	49 (45.79%)	107 (100%)	0.951
		71.6%	72.06%		
	Rural	23 (54.76%)	19 (45.24%)	42 (100%)	
		28.4%	27.94%		
Age at asthma diagnosis	<6 years old	38 (56.72%)	29 (43.28%)	67 (100%)	0.844
		46.91%	42.65%		
	6–11 years old	39 (52%)	36 (48%)	75 (100%)	
		48.15%	52.94%		
	12–17 years old	4 (57.14%)	3 (42.86%)	7 (100%)	
		4.94%	4.41%		

* Chi Square test. Values in gray are percentages summed by columns for each parameter.

**Table 3:** Distribution of the allergic pathologies associated with the study group.

Allergies	Category	COVID-19			*p* *
		Yes	No	Total	
		81 (54.36%)	68 (45.64%)	149 (100%)	
Allergic rhinitis	Yes	47 (50%)	47 (50%)	94 (100%)	0.162
		58.02%	69.12%		
	No	34 (61.82%)	21 (38.18%)	55 (100%)	
		41.98%	30.88%		
Atopic dermatitis	Yes	17 (68%)	8 (32%)	25 (100%)	0.133
		20.99%	11.76%		
	No	64 (51.61%)	60 (48.39%)	124 (100%)	
		79.01%	88.24%		
Food allergies	Yes	10 (76.92%)	3 (23.08%)	13 (100%)	0.087
		12.35%	4.41%		
	No	71 (52.21%)	65 (47.79%)	136 (100%)	
		87.65%	95.59%		
Pollen	Yes	15 (51.72%)	14 (48.28%)	29 (100%)	0.751
		18.52%	20.59%		
	No	66 (55%)	54 (45%)	120 (100%)	
		81.48%	79.41%		
House dust	Yes	18 (54.55%)	15 (45.45%)	33 (100%)	0.981
		22.22%	22.06%		
	No	63 (54.31%)	53 (45.69%)	116 (100%)	
		77.78%	77.94%		
Animal hair	Yes	8 (40%)	12 (60%)	20 (100%)	0.166
		9.88%	17.65%		
	No	73 (56.59%)	56 (43.41%)	129 (100%)	
		90.12%	82.35%		
Mold	Yes	12 (48%)	13 (52%)	25 (100%)	0.484
		14.81%	19.12%		
	No	69 (55.65%)	55 (44.35%)	124 (100%)	
		85.19%	80.88%		
Tobacco	Yes	1 (33.33%)	2 (66.67%)	3 (100%)	0.434
		1.23%	2.94%		
	No	80 (54.79%)	66 (45.21%)	146 (100%)	
		98.77%	97.06%		

* Chi Square test. Values in gray are percentages summed by columns for each allergy type.

**Table 4:** Distribution of the study group according to symptoms during the acute phase of COVID-19.

Parameter	Present (Number of Patients, Percent)
Fever	10 (12.35%)
Pharyngodynia	14 (17.28%)
Respiratory difficulties	13 (16.05%)
Cough	45 (55.56%)
Wheezing	26 (32.1%)
Thoracic constriction	2 (2.47%)
Myalgia	2 (2.47%)
Head aches	6 (7.41%)
Digestive manifestations	1 (1.23%)

**Table 5:** Distribution of the study group according to asthma evolution.

Parameter	Category	COVID-19			*p* *
		Yes	No	Total	
		81 (54.36%)	68 (45.64%)	149 (100%)	
Number of exacerbations	1/year	42 (72.41%)	16 (27.59%)	58 (100%)	<0.0005
		51.85%	23.53%		
	2/year	19 (61.29%)	12 (38.71%)	31 (100%)	
		23.46%	17.65%		
	3/year	7 (35%)	13 (65%)	20 (100%)	
		8.64%	19.12%		
	0/year	13 (32.5%)	27 (67.5%)	40 (100%)	
		16.05%	39.71%		
Compliance to controller therapy (parents/caregiver and/or self-reported)	Yes	53 (64.63%)	29 (35.37%)	82 (100%)	0.005
		65.43%	42.65%		
	No	28 (41.79%)	39 (58.21%)	67 (100%)	
		34.57%	57.35%		

* Chi Square test. Values in gray are percentages summed by columns for each parameter.

**Table 6:** Distribution of the study group according to asthma evolution and phenotype.

Parameter	Category	COVID-19			*p* *
		Yes	No	Total	
		81 (54.36%)	68 (45.64%)	149 (100%)	
FEV1/FVC Ratio	Normal	10 (20.83%)	38 (79.17%)	48 (100%)	<0.0005
		12.35%	55.88%		
	Variable airflow limitation	71 (70.3%)	30 (29.7%)	101 (100%)	
		87.65%	44.12%		
Asthma control at clinical visits	Well controlled	29 (39.19%)	45 (60.81%)	74 (100%)	<0.0005
		35.8%	66.18%		
	Partially controlled	52 (69.33%)	23 (30.67%)	75 (100%)	
		64.2%	33.82%		
Asthma phenotype	Allergic	47 (50.54%)	46 (49.46%)	93 (100%)	0.227
		58.02%	67.65%		
	Non-allergic	34 (60.71%)	22 (39.29%)	56 (100%)	
		41.98%	32.35%		

* Chi Square test. Values in gray are percentages summed by columns for each parameter.

**Table 7:** Distribution of the study group according to exhaled nitric oxide and ventilatory function.

Parameter	Category	COVID-19		*p* *
		Yes	No	
		81 (54.36%)	68 (45.64%)	
Nitric oxide **	Minimum	5	0	<0.0005
	Maximum	144	144	
	Median	30	14.5	
FVC ***	Minimum	0.60	0.46	0.069
	Maximum	1.22	1.27	
	Median	0.90	0.94	
FEV1 ***	Minimum	0.40	0.37	<0.0005
	Maximum	1.08	1.12	
	Median	0.78	0.88	
PEF ***	Minimum	0.35	0.37	0.466
	Maximum	1.08	1.32	
	Median	0.70	0.70	
FEF_25-75_ ***	Minimum	0.30	0.30	0.006
	Maximum	1.2	1.10	
	Median	0.70	0.82	
FEV1/FVC Ratio	Minimum	0.57	0.71	<0.0005
	Maximum	1.10	1.11	
	Median	0.88	0.92	

* Mann–Whitney U test; ** ppb = parts per billion; *** from predicted value.

**Table 8:** Distribution of the study group according to exhaled nitric oxide and ventilatory function, divided by cough duration.

Parameter	Category	Cough Duration			*p* *
		No Cough	<4 Weeks	>4 Weeks	
		8 (9.9%)	20 (24.7%)	53 (65.4%)	
Nitric oxide **	Minimum	7	5	6	<0.0005
	Maximum	55	48	114	
	Median	22.5	16.0	36.5	
FVC ***	Minimum	0.70	0.69	0.60	0.139
	Maximum	1.07	1.22	1.13	
	Median	0.90	0.98	0.88	
FEV1 ***	Minimum	0.40	0.63	0.49	0.048
	Maximum	0.88	1.08	0.95	
	Median	0.80	0.87	0.75	
PEF ***	Minimum	0.40	0.53	0.35	0.003
	Maximum	0.90	0.94	1.08	
	Median	0.75	0.79	0.65	
FEF_25-75_ ***	Minimum	0.40	0.5	0.3	0.100
	Maximum	1.0	1.2	1.2	
	Median	0.76	0.77	0.68	
FEV1/FVC Ratio	Minimum	0.57	0.79	0.66	0.234
	Maximum	0.97	1.10	1.0	
	Median	0.88	0.89	0.89	

* Kruskal–Wallis H test; ** ppb = parts per billion; *** from predicted value.

## Data Availability

The authors declare that the data of this research are available from the corresponding authors upon reasonable request.

## References

[1] GBD 2019 Diseases and Injuries Collaborators (2020). Global burden of 369 diseases and injuries in 204 countries and territories, 1990–2019: A systematic analysis for the Global Burden of Disease Study 2019. Lancet.

[2] Bush A. (2024). Evaluating Severe Therapy-Resistant Asthma in Children: Diagnostic and Therapeutic Strategies. Medicina.

[3] (2023). Global Strategy for Asthma Management and Prevention GINA. https://ginasthma.org/2023-gina-main-report/.

[4] Ioniuc I.K., Lupu A., Dragan F., Tarnita I., Alexoae M.M., Streanga V., Mitrofan C., Thet A.A., Nedelcu A.H., Salaru D.L. (2024). Oxidative Stress and Antioxidants in Pediatric Asthma’s Evolution and Management. Antioxidants.

[5] Murdoch J.R., Lloyd C.M. (2010). Chronic inflammation and asthma. Mutat. Res. Mol. Mech. Mutagen..

[6] Borish L., Culp J.A. (2008). Asthma: A syndrome composed of heterogeneous diseases. Ann. Allergy Asthma Immunol..

[7] Foppiano F., Schaub B. (2023). Childhood asthma phenotypes and endotypes: A glance into the mosaic. Mol. Cell Pediatr..

[8] Miculinić A., Mrkić Kobal I., Kušan T., Turkalj M., Plavec D. (2024). Current Challenges in Pediatric Asthma. Children.

[9] Pines J.M., Zocchi M.S., Black B.S., Carlson J.N., Celedon P., Moghtaderi A., Venkat A., US Acute Care Solutions Research Group (2021). Characterizing Pediatric Emergency Department Visits during the COVID-19 Pandemic. Am. J. Emerg. Med..

[10] Schuchat A., Team C.C.-R. (2020). Public Health Response to the Initiation and Spread of Pandemic COVID-19 in the United States, February 24–April 21, 2020. MMWR Morb. Mortal. Wkly. Rep..

[11] Bellino S., Rota M.C., Riccardo F., Andrianou X., Mateo Urdiales A., Del Manso M., Punzo O., Bella A., Villani A., Pezzotti P. (2021). Pediatric COVID-19 Cases Prelockdown and Postlockdown in Italy. Pediatrics.

[12] Vogel M., Beger C., Gausche R., Jurkutat A., Pfaeffle R., Korner A., Meigen C., Poulain T., Kiess W. (2021). COVID-19 pandemic and families’ utilization of well-child clinics and pediatric practices attendance in Germany. BMC Res. Notes.

[13] Cui X., Zhao Z., Zhang T., Guo W., Guo W., Zheng J., Zhang J., Dong C., Na R., Zheng L. (2021). A systematic review and meta-analysis of children with coronavirus disease 2019 (COVID-19). J. Med. Virol..

[14] Machado M.B., Fajardo T.C.G., de Oliveira L.B., de Quadros Junior A.C., Catalan D.T., Piovesan K.C., Garcia M.E.D., da Silva M.F., Dezena R.C.A.B., Passos S.D. (2022). Mild and Asymptomatic Coronavirus Disease in Children, Adolescents, and Household Contacts and Prolonged Viral Excretion. Int. J. Microbiol..

[15] Parisi G.F., Brindisi G., Indolfi C., Diaferio L., Marchese G., Ghiglioni D.G., Zicari A.M., del Giudice M.M. (2020). Upper airway involvement in pediatric COVID-19. Pediatr. Allergy Immunol..

[16] Indolfi C., Klain A., Dinardo G., D’Addio E., Ferrara S., Decimo F., Ciprandi G., Tosca M.A., Miraglia del Giudice M. (2024). COVID-19 Pediatric Follow-Up: Respiratory Long COVID-Associated Comorbidities and Lung Ultrasound Alterations in a Cohort of Italian Children. Children.

[17] Borch L., Holm M., Knudsen M., Ellermann-Eriksen S., Hagstroem S. (2022). Long COVID symptoms and duration in SARS-CoV-2 positive children—A nationwide cohort study. Eur. J. Pediatr..

[18] Esposito S., Principi N., Azzari C., Cardinale F., Di Mauro G., Galli L., Gattinara G.C., Fainardi V., Guarino A., Lancella L. (2022). Italian intersociety consensus on management of long covid in children. Ital. J. Pediatr..

[19] Choi J.H., Choi S.H., Yun K.W. (2022). Risk Factors for Severe COVID-19 in Children: A Systematic Review and Meta-Analysis. J. Korean Med. Sci..

[20] Costa V.C.d., Montarroyos U.R., Lopes K.A.d.M., Santos A.C.O.d. (2024). Severity Profile of COVID-19 in Hospitalized Pediatric Patients. Children.

[21] Abrams E.M., 't Jong G.W., Yang C.L. (2020). Asthma and COVID-19. CMAJ.

[22] Shaker M.S., Oppenheimer J., Grayson M., Stukus D., Hartog N., Hsieh E.W.Y., Rider N., Dutmer C.M., Vander Leek T.K., Kim H. (2020). COVID-19: Pandemic Contingency Planning for the Allergy and Immunology Clinic. J. Allergy Clin. Immunol. Pract..

[23] Robert Koch Institut. https://www.rki.de/EN/Content/infections/epidemiology/inf_dis_Germany/COVID-19/Long-COVID/content-total.html.

[24] National Institute of Health (2023). Long COVID Resources and Research About Long COVID. https://www.nhlbi.nih.gov/covid/long-covid#:~:text=Long%20COVID%2C%20long%2Dhaul%20COVID,than%20symptoms%20of%20COVID%2D19%20.

[25] World Health Organization. https://www.who.int/publications/i/item/WHO-2019-nCoV-Post_COVID-19_condition-Clinical_case_definition-2021.1.

[26] Pazukhina E., Andreeva M., Spiridonova E., Bobkova P., Shikhaleva A., El-Taravi Y., Rumyantsev M., Gamirova A., Bairashevskaia A., Petrova P. (2022). Prevalence and risk factors of post-COVID-19 condition in adults and children at 6 and 12 months after hospital discharge: A prospective, cohort study in Moscow (StopCOVID). BMC Med..

[27] Crook H., Raza S., Nowell J., Young M., Edison P. (2021). Long COVID-mechanisms, risk factors, and management. BMJ.

[28] Leung N.H.L., Chu D.K.W., Shiu E.Y.C., Chan K.H., McDevitt J.J., Hau B.J.P., Yen H.L., Li Y., Ip D.K.M., Peiris J.S.M. (2020). Respiratory virus shedding in exhaled breath and efficacy of face masks. Nat. Med..

[29] Papadopoulos N.G., Christodoulou I., Rohde G., Agache I., Almqvist C., Bruno A., Bonini S., Bont L., Bossios A., Bousquet J. (2011). Viruses and bacteria in acute asthma exacerbations--a GA² LEN-DARE systematic review. Allergy.

[30] Kenyon C.C., Hill D.A., Henrickson S.E., Bryant-Stephens T.C., Zorc J.J. (2020). Initial effects of the COVID-19 pandemic on pediatric asthma emergency department utilization. J. Allergy Clin. Immunol. Pract..

[31] Krivec U., Kofol Seliger A., Tursic J. (2020). COVID-19 lockdown dropped the rate of paediatric asthma admissions. Arch. Dis. Child..

[32] Papadopoulos N.G., Mathioudakis A.G., Custovic A., Deschildre A., Phipatanakul W., Wong G., Xepapadaki P., Abou-Taam R., Agache I., Castro-Rodriguez J.A. (2021). Childhood asthma outcomes during the COVID-19 pandemic: Findings from the PeARL multi-national cohort. Allergy.

[33] Liu S., Zhi Y., Ying S. (2020). COVID-19 and asthma: Reflection during the pandemic. Clin. Rev. Allergy Immunol..

[34] Liuzzo Scorpo M., Ferrante G., La Grutta S. (2021). An Overview of Asthma and COVID-19: Protective Factors Against SARS-COV-2 in Pediatric Patients. Front. Pediatr..

[35] Seidman M.D., Gurgel R.K., Lin S.Y., Schwartz S.R., Baroody F.M., Bonner J.R., Dawson D.E., Dykewicz M.S., Hackell J.M., Han J.K. (2015). AAO-HNSF. Clinical practice guideline: Allergic rhinitis. Otolaryngol. Head. Neck Surg..

[36] Bousquet J., Anto J.M., Bachert C., Baiardini I., Bosnic-Anticevich S., Walter Canonica G., Melén E., Palomares O., Scadding G.K., Togias A. (2020). Allergic rhinitis. Nat. Rev. Dis. Primers.

[37] Skoner D.P. (2001). Allergic rhinitis: Definition, epidemiology, pathophysiology, detection, and diagnosis. J. Allergy Clin. Immunol..

[38] Meltzer E.O., Blaiss M.S., Derebery M.J., Mahr T.A., Gordon B.R., Sheth K.K., Simmons A.L., Wingertzahn M.A., Boyle J.M. (2009). Burden of allergic rhinitis: Results from the Pediatric Allergies in America survey. J. Allergy Clin. Immunol..

[39] Schuler Iv C.F., Montejo J.M. (2019). Allergic Rhinitis in Children and Adolescents. Pediatr. Clin. N. Am..

[40] Peat J.K., Salome C.M., Woolcock A.J. (1990). Longitudinal changes in atopy during a 4-year period: Relation to bronchial hyperresponsiveness and respiratory symptoms in a population sample of Australian schoolchildren. J. Allergy Clin. Immunol..

[41] Cheng M., Dai Q., Liu Z., Wang Y., Zhou C. (2024). New progress in pediatric allergic rhinitis. Front. Immunol..

[42] Rayner D.G., Wang E., Su C., Patel O.D., Aleluya S., Giglia A., Zhu E., Siddique M. (2024). Risk factors for long COVID in children and adolescents: A systematic review and meta-analysis. World J. Pediatr..

[43] Mazzone S.B. (2023). Neurobiology of Coughing in Children. J. Clin. Med..

[44] Chung K.F., McGarvey L., Song W.J., Chang A.B., Lai K., Canning B.J., Birring S.S., Smith J.A., Mazzone S.B. (2022). Cough hypersensitivity and chronic cough. Nat. Rev. Dis. Primers.

[45] Liu L., Zhang L., Zhou P., Zhou W., Li L., Zeng L., Li N., Zhao R., Han T. (2024). Cough symptoms in children following COVID-19: A single-center retrospective study. Front. Pediatr..

[46] Sansone F., Di Filippo P., Russo D., Sgrazzutti L., Di Pillo S., Chiarelli F., Attanasi M. (2024). Lung functionassessment in children with Long-Covid syndrome. Pediatr. Pulmonol..

[47] Li W., Moore M.J., Vasilieva N., Sui J., Wong S.K., Berne M.A., Somasundaran M., Sullivan J.L., Luzuriaga K., Greenough T.C. (2003). Angiotensin-converting enzyme 2 is a functional receptor for the SARS coronavirus. Nature.

[48] Denina M., Pruccoli G., Scolfaro C., Mignone F., Zoppo M., Giraudo I., Silvestro E., Bertolotti L., Rosati S., Ramenghi U. (2020). Sequelae of COVID-19 in Hospitalized Children: A 4-Months Follow-Up. Pediatr. Infect. Dis. J..

[49] Radtke T., Ulyte A., Puhan M.A., Kriemler S. (2021). Long-term Symptoms After SARS-CoV-2 Infection in Children and Adolescents. JAMA.

[50] Nassoro D.D., Mujwahuzi L., Mwakyula I.H., Possi M.K., Lyantagaye S.L. (2021). Asthma and COVID-19: Emphasis on Adequate Asthma Control. Can. Respir. J..

[51] Agondi R.C., Menechino N., Marinho A.K.B.B., Kalil J., Giavina-Bianchi P. (2024). Worsening of asthma control after COVID-19. Front. Med..

[52] Dweik R.A., Boggs P.B., Erzurum S.C., Irvin C.G., Leigh M.W., Lundberg J.O., Olin A.C., Plummer A.L., Taylor D.R. (2011). American Thoracic Society Committee on Interpretation of Exhaled Nitric Oxide Levels (FENO) for Clinial Applications. An official ATS clinical practice guideline: Interpretation of exhaled nitric oxide levels (FeNO) for clinical applications. Am. J. Respir. Crit. Care Med..

[53] Amat F., Labbé A. (2018). Biomarkers for severe allergic asthma in children: Could they be useful to guide disease control and use of omalizumab?. Expert. Rev. Respir. Med..

[54] Wang Z., Pianosi P.T., Keogh K.A., Zaiem F., Alsawas M., Alahdab F., Almasri J., Mohammed K., Larrea-Mantilla L., Farah W. (2018). The diagnostic accuracy of fractional exhaled nitric oxide testing in asthma: A systematic review and meta-analyses. Mayo Clin. Proc..

[55] Barreto M., Evangelisti M., Montesano M., Martella S., Villa M.P. (2020). Pulmonary Function Testing in Asthmatic Children. Tests to Assess Outpatients During the Covid-19 Pandemic. Front. Pediatr..

[56] Cameli P., Bargagli E., Bergantini L., d'Alessandro M., Giugno B., Gentili F., Sestini P. (2021). Alveolar Nitric Oxide as a Biomarker of COVID-19 Lung Sequelae: A Pivotal Study. Antioxidants.

[57] Zhao Y.M., Shang Y.M., Song W.B., Li Q.Q., Xie H., Xu Q.F., Jia J.L., Li L.M., Mao H.L., Zhou X.M. (2020). Follow-up study of the pulmonary function and related physiological characteristics of COVID-19 survivors three months after recovery. eClinicalMedicine.

[58] Patil S., Teli A., Nayaka R., Patil P. (2024). Assessment of Pulmonary Function in COVID-19 Recovered Health Science Students: A Descriptive Cross-sectional Study. J. Clin. Diag. Res..

[59] Rao D.R., Gaffin J.M., Baxi S.N., Sheehan W.J., Hoffman E.B., Phipatanakul W. (2012). The utility of forced expiratory flow between 25% and 75% of vital capacity in predicting childhood asthma morbidity and severity. J. Asthma.

[60] Kanchongkittiphon W., Gaffin J.M., Kopel L., Petty C.R., Bollinger M.E., Miller R.L., Perzanowski M., Matsui E.C., Phipatanakul W. (2016). Association of FEF25%-75% and bronchodilator reversibility with asthma control and asthma morbidity in inner-city children with asthma. Ann. Allergy Asthma Immunol..

[61] Simon M.R., Chinchilli V.M., Phillips B.R., Sorkness C.A., Lemanske R.F., Szefler S.J., Taussig L., Bacharier L.B., Morgan W., Childhood Asthma Research and Education Network of the National Heart, Lung, and Blood Institute (2010). Forced expiratory flow between 25% and 75% of vital capacity and FEV1/forced vital capacity ratio in relation to clinical and physiological parameters in asthmatic children with normal FEV1 values. J. Allergy Clin. Immunol..

[62] Onay Z.R., Oksay S.C., Mavi Tortop D., Bilgin G., Ayhan Y., Durankus F., Girit S. (2024). Impact of Long COVID on Lung Function in Children. Medeni. Med. J..

[63] Salem A.M., Al Khathlan N., Alharbi A.F., Alghamdi T., AlDuilej S., Alghamdi M., Alfudhaili M., Alsunni A., Yar T., Latif R. (2021). The Long-Term Impact of COVID-19 Pneumonia on the Pulmonary Function of Survivors. Int. J. Gen. Med..

[64] Torres-Castro R., Vasconcello-Castillo L., Alsina-Restoy X., Solis-Navarro L., Burgos F., Puppo H., Vilaró J. (2021). Respiratory function in patients post-infection by COVID-19: A systematic review and meta-analysis. Pulmonology.

[65] Chen X., Han P., Kong Y., Shen K. (2024). The relationship between changes in peak expiratory flow and asthma exacerbations in asthmatic children. BMC Pediatr..

[66] Dondi A., Calamelli E., Piccinno V., Ricci G., Corsini I., Biagi C., Lanari M. (2017). Acute Asthma in the Pediatric Emergency Department: Infections Are the Main Triggers of Exacerbations. BioMed Res. Int..

[67] Halm E.A., Mora P., Leventhal H. (2006). No symptoms, no asthma: The acute episodic disease belief is associated with poor self-management among inner-city adults with persistent asthma. CHEST.

[68] Feldman J.M., Serebrisky D., Starr S., Castaño K., Greenfield N., Silverstein G., Fruchter N., Mammen J., McGovern C., Arcoleo K. (2023). Reduced asthma morbidity during COVID-19 in minority children: Is medication adherence a reason?. J. Asthma.

